# Predictors of sexual abstinence among Wolaita Sodo University Students, South Ethiopia

**DOI:** 10.1186/1742-4755-10-18

**Published:** 2013-04-01

**Authors:** Terefe Gelibo, Tefera Belachew, Tizita Tilahun

**Affiliations:** 1 Department of Public Health, Wolaita Sodo University, Sodo, South Ethiopia; 2 Departments of Population and Family Health, Jimma University, Jimma, Ethiopia

**Keywords:** Sexual, Abstinence, Wolaita Sodo, University, Students Ethiopia

## Abstract

**Background:**

It is over 30 years since the first case of AIDS [Acquired Immune Deficiency Syndrome] was identified. Attention has been focused recently on the promotion of the “ABCs” of HIV prevention (being abstinent or delaying sex, remaining faithful to one sexual partner, and using condoms consistently during sexual intercourse). As programs that focus on ABCs to prevent heterosexual transmission HIV are rolled out, questions of how well university students who come from varied cultural contexts actually understand the terms and address challenges to adopt behaviors is unanswered. In Ethiopia, despite the mushrooming number of students in the higher learning institutions with the current figure being 210,000 students accommodated in 33 public and 72 private higher learning institutions, sexual and reproductive health services, are not delivered in an organized way. The objective of this study is to identify factors associated with Sexual abstinence among Wolaita Sodo University students to provide evidence for designing appropriate interventions.

**Methods:**

A Cross-Sectional study was conducted among 750 undergraduate students selected from Wolaita Sodo University using a stratified simple random sampling technique during the academic year. Data were collected using structured self administered questionnaire, focus group discussion and in depth interview guides as tools for data collection. Ethical clearance was obtained from Jimma University and informed consent was obtained from the participants after explaining purpose of study. Statistical tests were employed wherever necessary at the significance level of 0.05.

**Results:**

All of the participants had heard about HIV/AIDS of which 97.3% had good knowledge. Higher proportions of male students were sexually active than their counter parts. Students with better knowledge on HIV AIDS were 6.6 (95%CI=1.6, 12.9) times more likely to abstain from sexual intercourse than their counter parts.

**Conclusions:**

Knowledge of students about risk of HIV infection is strong predictors of sexual abstinence of students which were less observed among students who came from rural areas. The university needs to intensify strong behavior change communication using multiple strategies through the active involvement of students themselves within the university’s premises and in the surrounding community in collaboration with stakeholders to promote Sexual abstinence.

## Introduction

It has been more than 3 decades since the first cases of AIDS [Acquired Immune Deficiency Syndrome] were identified [1]. HIV pandemic remains the most serious of infectious disease challenges to public health. Although there is still neither a vaccine nor cheap, assured and effective treatment for HIV/AIDS, attention has been focused recently on the promotion of the “ABCs” of HIV prevention (being abstinent or delaying sex, remaining faithful to one sexual partner, and using condoms consistently during sexual intercourse [1-3]. The disease deprives countries, communities, and households of their strong, productive people [1]. Currently more than 33.2 million people were living with the virus worldwide and Africa, and in particular Sub-Saharan Africa, is at present in the “eye of the storm” [1,2,4].

In Ethiopia, HIV epidemic has penetrated even the hard-to-reach rural areas. However, with the existing socio-cultural diversity of Ethiopia, the pattern and distribution of HIV widely varies by region, place of residence and sex [5]. Even though the virus affects all social sectors of the population, the epidemic among adolescents is the fastest growing partly because of young people’s vulnerability and because of low use of preventive services [6]. The young never married females carry the greatest risk of HIV infection because of both biological, cultural factors. Often they form sexual relationships with men who are on average ten years older and as the girls are unlikely to have had any training or experience in sexual negotiation skills, and are especially vulnerable in situations with older men where age, wealth, physical strength and other power dynamics put them at a disadvantage [5,7]. The EDHS 2005 confirmed the widely-held belief that girls start having sex earlier than boys [5].

Universities in the high prevalence countries of Sub Saharan Africa significant proportion of their students and staff may be infected with HIV [8]. Therefore, universities are one of the priority targets for promoting and enhancing healthy sexual behavior among students, who are the future human capital of the nation [8,9]. University students are youths who are vulnerable, may not have adequate or consistent income, and are living without consistent adult supervision for first time in their lives. All these compounded with peer pressure makes them highly vulnerable to HIV [8]. Unprotected sexual practice patterns among university students lags behind knowledge and attitude towards prevention of STDs and condom use [7]. A number of studies have showed that HIV/AIDS risk behaviors have progressively been on increase and being the problem of students in the higher education institutions in Ethiopia [10-12]. In general, students are one part of the most vulnerable groups in the country particularly college and pre-college students: in recent years a number of new training and higher learning institutions have been opened. These sites and their student populations have not been studied, but there is some circumstantial evidence suggesting the existence of widespread unsafe sexual practices [13]. After joined UN Member States in 2006 Ethiopia issued the Political Declaration on HIV/AIDS, which included a commitment to move towards the goal of universal access to HIV prevention, treatment, care and support by 2010. All schools including colleges and Universities will have HIV/AIDS information centers [14]. In Wolaita Sodo University HIV/AIDS prevention activities were not organized and the budget source is from external through project based grants and which is not consistent. The objective of this study is to identify predictors of sexual abstinence among WSU students.

## Methods

### Study setting and sample

A cross-sectional study was conducted in Wolaita Sodo University from April 20 to 24, 2010. Wolaita Sodo University is one of the public universities in Ethiopia. The University has total of 4246 regular undergraduate students.

### Sample size and sampling procedure

The sample size was determined taking 66.8% expected prevalence for sexual abstinence reported by the study among Jimma university students (10), assuming 5% margin of error and 95% confidence level, design effect of 2 and 10% for non response rate. The calculated sample size was 750. To come up with the size of respondent first all faculties in Wolaita Sodo University were stratified into health and non health faculties and then students were selected using stratified sampling with proportional allocation to sample size (PAS). Number of students in each study year within the departments was selected using the principle of proportional to size allocation method. Then, students were selected from each department by simple random sampling technique using lists of students as sampling frame.

### Measurement

Data were collected using self administered structured questionnaire under the supervision of the principal investigator, focus group discussion and in-depth interviews were also conducted to generate qualitative data that can enable to have deeper understanding of the behaviors. Seven Focus Group Discussions (FGDs) (3 with males and 4 with female students) were conducted with each consisting of 6–12 students. The data were collected on the dependent variable i.e. Sexual abstinence and the independent variables including socio demographic and socioeconomic variables, knowledge related to HIV/AIDS transmission and prevention and communication with parents about sexual issues.

### Statistical analysis

The data were coded, edited and entered, cleaned and analyzed using SPSS for windows version 16. Statistical analysis has three steps: first association was done between potential predictors of sexual abstinence use using Pearson’s chi-square and 95% confidence intervals were calculated to show bivariate association. Next, to identify the independent contribution of each variable on sexual abstinence, multivariable logistic regression models were used. Adjusted odds ratio, and confidence intervals were calculated to assess the association between independent variables and sexual abstinence. Finally, it was evaluated that variables identified as associated (P<0.05) with sexual abstinence in the multivariable logistic regression analysis were used. The qualitative data were transcribed into English language verbatim, read critically and essential themes were identified. Then ideas related to each team were color coded. Then ideas were organized into concepts and presented using narratives. The result is presented in tri-angulations with the quantitative survey using subjects verbatim as illustrations.

### Data quality control

The collected data were reviewed and checked for completeness and relevance by the supervisors and principal investigator each day and was checked, coded and entered in to computer and cleaned before analysis. Investigators discussion guide was cross checked between the note takers and the principal investigator then transcribed into hand written English transcript

### Ethical issues

Ethical clearance was obtained from Jimma University, College of Public Health and Medical science; a letter of cooperation was sent from Population and Family Health Department to the study area (Wolaita Sodo University). Informed consent was obtained from each study subjects prior to the administration of questionnaire after the purpose of the study was explained to respondent. Confidentiality was maintained by omitting their personally identifiable information such as names from the questionnaire.

## Results

Most of the selected students participated in the survey (749 out of 750 students) making a response rate of 99.8%. Males constitute 77.8%. Most of students were living on campus. The mean (±SD) age was 20.9 (±1.5) years. Amhara and orthodox Christian were the predominant ethnicities and religions, respectively (Table 1). Concerning substance use, 32.7% of the students drank alcohol of which 65.6% drunk alcohol from time to time, 27.9 tried once or twice and 6.6% drunk alcohol daily. Similarly, 13.2% of students smoked cigarettes, while 24.2% chewed chat. There was significant association between frequency of alcohol intake and sexual activity. Students who take alcohol daily were 9.2 (95% CI 5.7, 12.1) times more likely to be engaged in sexual intercourse than those who tried alcohol once or twice.

**Table 1:** Socio-demographic characteristics of Wolaita Sodo University students, June,2010

**Socio demographic and socio economic characteristics**	**Number**	**Percent**
**Sex**	583	77.8
Male	166	22.2
Female		
**Ethnicity**	181	24.2
Amhara	129	17.2
Hadiya	128	17.1
Oromo	101	13.5
Gurage	86	11.5
Wolaita	52	6.9
Tigre	72	9.5
Others*		
**Religion**	368	49.1
Orthodox Christian	107	14.3
Muslim	261	34.8
Protestant	13	1.8
Others **	194	25.9
**Faculty or school**	154	20.6
Natural Science	144	19.2
Business economics	103	13.8
Social Science	76	10
Agriculture	42	5.6
Health Science	36	4.6
School of Technology		
School of Law		
**Academic year**	356	47.5
Year I	153	20.4
Year II	240	32.0
Year III		
**Current place of accommodation**	733	97.9
Dormitory	13	1.7
With parents	3	0.4
Outside campus rented		
**Self reported academic performance**	250	33.4
Below average	382	51.0
At least average	117	15.6
Ten top		

*Kembata, Dawro, Gamo, Sidama ** Catholic, Jehovah

### Sexual practice

Around 53.3% of respondents ever had sexual intercourse in their lifetime among these, 25.8% had their first sex after they joined University (Table 2). Female students were 3.7 (95% CI 3.1, 4.4) times more likely to start their first sexual intercourse after joining university than male students and larger proportion (40.8%) practiced their first sex during the third years compared with 34.0% who had it during the first year. And also focus group discussants added that most of students perceive sexual initiation at campus as the most enjoyable part of university life.

**Table 2:** Sexual practice among students of Wolaita Sodo University, 2010

**Variables**		**Number**	**Percent**
**Sexual experience (n=749)**	Yes	399	53.3
	No	350	46.7
**Sexual intercourse in the last 12 months (n=399)**	Yes	244	61.2
	No	155	38.8
**Age you started first sexual intercourse (n=399)**	<18 years	60	15.0
	≥18 years	399	85.0
**Where did you start first sex (n=399)**	Elementary school	28	7.0
	High school	267	62.0
	University	104	22.2
**Sexual intercourse in the last 6 months (n=399)**	Yes	218	54.6
	No	181	45.4
	No	198	49.6
**Ever had sexually transmitted infections (n=398)**	Yes	112	28.1%
	No	286	71.9
**Ever had sex with CSWs (n=338)**	Yes	84	24.9
	No	254	75.1
**Life time number of sexual partners (n=201)**	One	59	29.4
	More than one	142	70.6
**Have you ever had sex after alcohol use (n=399)**	Yes	214	53.6
	No	185	46.4

The mean age of first sexual experience was 18.4(±1.75) and 19.9(±1.87) for males and females, respectively. Male students were 2.2 (95%CI 1.1, 4.8) times more likely to start first sexual intercourse below the age of 18 years than female students. With regards to parent adolescent communication about sexual issues, the findings indicated that 69.8% had better communication with their parents about sexual issues. There is no significant gender difference (70% male vs 69.3% females) in participants’ communication with their parents about sexual issues. Focus group discussants claimed that sexuality is seen as a taboo in most of “Ethiopian cultures”. The most common reasons mentioned for remaining abstinent were: wanted to wait until one is older or married, concerns of risk of pregnancy and disease and religious values (Table 3).

**Table 3:** Percentage distribution of Wolaita Sodo University students, who have never had intercourse, by primary reason for remaining sexually abstinent June, 2010

**Reason**	**Percent**	**Males (N=278)**	**Females (N=72)**
I want to wait until I’m older or married	30.0%	23.7%*	52.2%
My religious values are against it.	37.7%	36.3%*	41.1%
I don’t want the risk of pregnancy or disease.	23.1%	29.1%*	2.0%
My parents’ values are against it.	2.6%	3.2%*	2.0%
Others**	6.6%	7.6%	2.8%

** Others include cultures, friends approval etc.

*Difference between males and females is significant at p≤.001.

### Knowledge on HIV/AIDS transmission and prevention and HIV*/AIDS Risk Perception*

With regards to Knowledge on HIV/AIDS and HIV/AIDS risk perception, all of the participants had heard about HIV/AIDS of whom 97.3% had good knowledge with an overall Knowledge score of 60% and above out of 100. Levels of HIV/AIDS-related knowledge were similar between sexes. The results indicated that students of various faculties did not have the same knowledge about HIV/AIDS. Students of Faculty of Health Sciences had the highest (Mean= 10.99) where as faculty of Agriculture (Mean= 9.73) had the least. Concerning the participants risk perception of HIV/AIDS, 48.9% perceive that they were at risk of HIV infection. There were gender differences in HIV risk perception. Female students were 1.7 (95% CI 1.20, 2.43) times more likely to report low perception to HIV than male students.

### Factors associated with sexual abstinence

Bivariate and multivariable logistic regression analysis was used to calculate odds ratios and corresponding 95% confidence intervals for the predictors of Sexual abstinence. In the bivariate analysis alcoholic non intake, family living arrangement, AIDS risk perception, place of previous residence, level of Knowledge on HIV/AIDS, self reported academic performance, frequency of church/mosque attendances and communication with parents about sexual issues were found to be associated with sexual abstinence (Table 4).

**Table 4:** Unadjusted and adjusted odds ratio and (95% confidence intervals) of logistic regression showing effects of selected Characteristics (predictor variables) on the likely hood of sexual abstinence among Wolaita Sodo University students

**Predictor variables of sexual abstinence**		**Ever had sexual intercourse**		**Crude OR(95%CI)**	**Adjusted OR (95.0% C.I)**
		**No (%)**	**Yes (%)**		
Sex	Male	37.1	40.7	1.2 (0.8,1.7)	0.9(0.6,1.6)
	Female	9.6	12.6	1.00	1.00
Drink Alcohol (N=749)	No	41.3	26.0	7.9 (5.4,11.5)*	6.2 (3.8,10.9)*
	Yes	5.4	27.3	1.00	1.00
AIDS risk perception (N=749)	No	11.8	39.3	1.00	1.00
	Yes	34.9	14.0	4.1(3.0,5.6)*	2.6 (1.7,4.1)*
Place of resident (N=749)	Rural	36.3	17.1	1.6 (1.2,2.2)*	2.1 (1.3,3.2)*
	Urban	13.4	36.2	1.00	1.00
Level of knowledge on HIV (N=749).	Poor	1.0	1.7	1.00	1.00
	Better	45.7	51.6	3.6 (1.2,10.9)*	6.6 (1.6,12.9)*
Self reported Academic performance (N=749)	Below average	4.6	28.8	1.00	1.00
	At least average	42.1	24.5	3.8 (2.7,5.3)*	2.9 (1.9,4.6)*
Frequency of church attendance (N=749)	≤ twice a month	5.4	34.1	1.00	1.00
	>twice a month	41.3	19.2	14.8 (10.1,19.5)*	6.4 (4.1,9.9)*
Communication with parents about sexual issues (N=749)	Poor	8.7	21.5	1.00	1.00
	Better	38.0	31.8	2.3 (1.7,3.2)*	1.6 (1.1,2.5)*

*Statistically significant at P< 0.05.

In multivariable logistic regression analysis after adjusted for potential confounders in the final model, alcoholic non intake, high AIDS risk perception, being rural place of previous residence, having good level of Knowledge on HIV/AIDS, Self reported academic performance of at least average, increased frequency of church/mosque attendances and having good communication with parents about sexual issues were the potent predictors of sexual abstinence. Students who came to the University from rural areas(AOR=2.1), do not take alcohol[AOR=6.2], those with better knowledge on HIV AIDS[A=6.6], high AIDS risk perception[AOR=2.6], self reported academic performance of at least average and above[AOR=2.9], attending church more than 2 times a month(AOR=6.4] and better communication with parents about sexual issues(AOR=1.6) were times more likely to abstain from sexual intercourse than their counter parts respectively (Table 4).

## Discussion

The predictors of sexual abstinence have been seen comprehensively both quantitatively and qualitatively and our findings showed that socio demographic and economic, behavioral and psychosocial characteristics were found to have an association. Higher proportions of male university students were sexually active than female students this fact was consistent with a number of other studies done in different parts of the country [11,15,16]. For instance, a survey done in Gondar College of medical science [11], [55.2% VS 8.3%], a study conducted among university students in china [16] where 17.6% of males and 8.6% of females were sexually active. Another study done in Haramaya University [15] showed similar evidence [59.4% vs. 38.9%].

In this study gender was not significantly associated with sexual abstinence among university students. Level of knowledge among participants was one of the potent predictors of sexual abstinence. This contradicts with a study among urban university students in Philippines indicated that gender was one of the predictor variables of sexual abstinence [17]. The possible explanation for the difference may be due to the difference in the study area that in this study finding abstinence among students vary by place of 1^st^ sexual practice; females abstain from sexual intercourse before joining university.

Students who came to the University from rural areas, those with better knowledge on HIV AIDS, self reported academic performance of at least average and above and better communication with parents about sexual issues were [2.1, 6.6, 2.9, 1.6] times more likely to abstain from sexual intercourse than their counter parts respectively. These contradict with study among urban university students in Philippines where the above variables were not associated with likelihood of sexual abstinence [17]*.* The possible explanation for this difference may be the difference in the study areas, the samples in this study were from both urban and rural areas. The inclusion of students from both urban and rural areas in this study could have made differences in the predictor variables.

## Competing interests

The authors declare that they have no competing interests.

## Authors’ contributions

These authors (TG, TB, TT) contributed equally to this work. All authors read and approved the final manuscript.
